# Artificial Intelligence-Driven Digital Tools for Diabetes Self-management in Children: A Scoping Review

**DOI:** 10.5812/ijem-169084

**Published:** 2026-08-11

**Authors:** Maryam Nakhaee Moghadam, Sara Amini, Mojtaba Lotfi, Mehrnaz Nazari Rad, Jalal Nourmohammadi

**Affiliations:** 1 Children and Adolescents Health Research Center, Resistant Tuberculosis Institute, Zahedan University of Medical Sciences and Health Services, Zahedan, Iran; 2 Department of Pediatrics, Mashhad University of Medical Sciences, Mashhad, Iran; 3 Clinical Research Development Unit, Akbar Hospital, Faculty of Medicine, Mashhad University of Medical Sciences, Mashhad, Iran; 4 Student Research Committee, Faculty of Nursing and Midwifery, Zahedan University of Medical Sciences, Zahedan, Iran

**Keywords:** Diabetes Mellitus, Child, Self-management, Artificial Intelligence, Digital Health

## Abstract

**Context:**

Managing diabetes in children is challenging and requires continuous monitoring and structured self-care support. Artificial intelligence (AI)-driven digital tools may enhance self-management and improve health outcomes. This scoping review aimed to synthesize the existing evidence on AI-driven digital tools for pediatric diabetes self-management, focusing on their functionalities, benefits, and limitations.

**Evidence Acquisition:**

From an initial pool of 97 articles published between January 2010 and March 1, 2025, 19 studies met the predefined inclusion criteria. A systematic search was conducted in PubMed, Scopus, Web of Science, CINAHL, PsycINFO, and Embase using predefined keywords. Two independent reviewers performed screening and data extraction. Methodological quality was appraised using the Cochrane Risk of Bias Tool, the Newcastle-Ottawa Scale, and the Critical Appraisal Skills Programme (CASP) Checklist. Qualitative and quantitative findings were synthesized thematically.

**Results:**

The 19 included studies addressed several overlapping application domains of AI-driven digital tools for pediatric diabetes self-management. Overall, 8 studies focused primarily on glucose prediction, glycemic monitoring, or pattern recognition; 4 studies evaluated insulin dosing optimization and clinical decision-support systems; 6 studies investigated personalized education, behavioral support, user engagement, or self-management learning interventions; 5 studies examined telemedicine, remote monitoring, or healthcare access; and 5 studies primarily addressed ethical, methodological, transparency, or implementation challenges.

**Discussion:**

AI-based digital tools have considerable potential to support diabetes self-management in children. Future research should prioritize long-term evaluations, the inclusion of diverse populations, and rigorous assessments of clinical outcomes, data privacy, and algorithm performance.

## 1. Introduction

Diabetes in children, encompassing both type 1 (T1D) and type 2 (T2D), presents distinct yet overlapping challenges in daily self-management (1). Children with T1D must carefully monitor blood glucose, follow dietary guidelines, engage in regular physical activity, and administer insulin accurately (2). In T2D, management relies largely on lifestyle modifications and, in some cases, medications or insulin (3). Effective self-care can be challenging for children because of limited cognitive development, evolving decision-making skills, and fluctuating motivation. Young patients with T1D often depend on parental guidance for insulin dosing and glucose monitoring, whereas children with T2D may find sustaining lifestyle changes and adhering to treatment particularly demanding (4).

Poor management of either form of diabetes can result in acute complications, long-term organ damage, reduced quality of life, and increased psychological and financial strain on families. Research indicates that structured interventions, such as family-focused education, behavioral support, and personalized self-management programs, can substantially improve adherence and clinical outcomes in pediatric diabetes (5). Artificial intelligence has recently emerged as a promising approach to enhance self-management in children with diabetes. AI-driven digital tools, including mobile applications, wearable devices, remote monitoring platforms, and intelligent decision-support systems, enable the continuous collection and analysis of complex data, such as glucose measurements, physical activity, and dietary intake (6). Machine learning (ML) algorithms and predictive models can identify intricate patterns in glucose fluctuations and provide personalized, actionable feedback to support daily decision-making. These tools not only improve glycemic control but also promote active engagement and self-efficacy in children while enabling parents to provide informed and targeted support (7).

Despite the rapid development of AI-based interventions, systematic and comprehensive evidence regarding their application in pediatric populations remains limited. Most existing studies focus on specific tools or isolated aspects of self-management and therefore fail to provide a holistic overview of AI’s potential to empower children to manage their disease effectively (8). This research gap constrains healthcare professionals, developers, and policymakers from implementing evidence-based, child-centered solutions that maximize the benefits of AI (9). Given the clinical importance of pediatric self-management, the rapid growth of AI technologies, and the current lack of comprehensive synthesis, this scoping review is both timely and essential. The findings are expected to inform researchers, technology developers, and healthcare policymakers in designing intelligent, personalized, and child-centered interventions that improve self-management and clinical outcomes in pediatric diabetes.

The aim of this study was to systematically identify, categorize, and evaluate AI-driven digital tools for diabetes self-management in children, highlighting emerging trends, benefits, limitations, and gaps in current knowledge.

## 2. Methods

### 2.1. Overview

**Figure 1.**
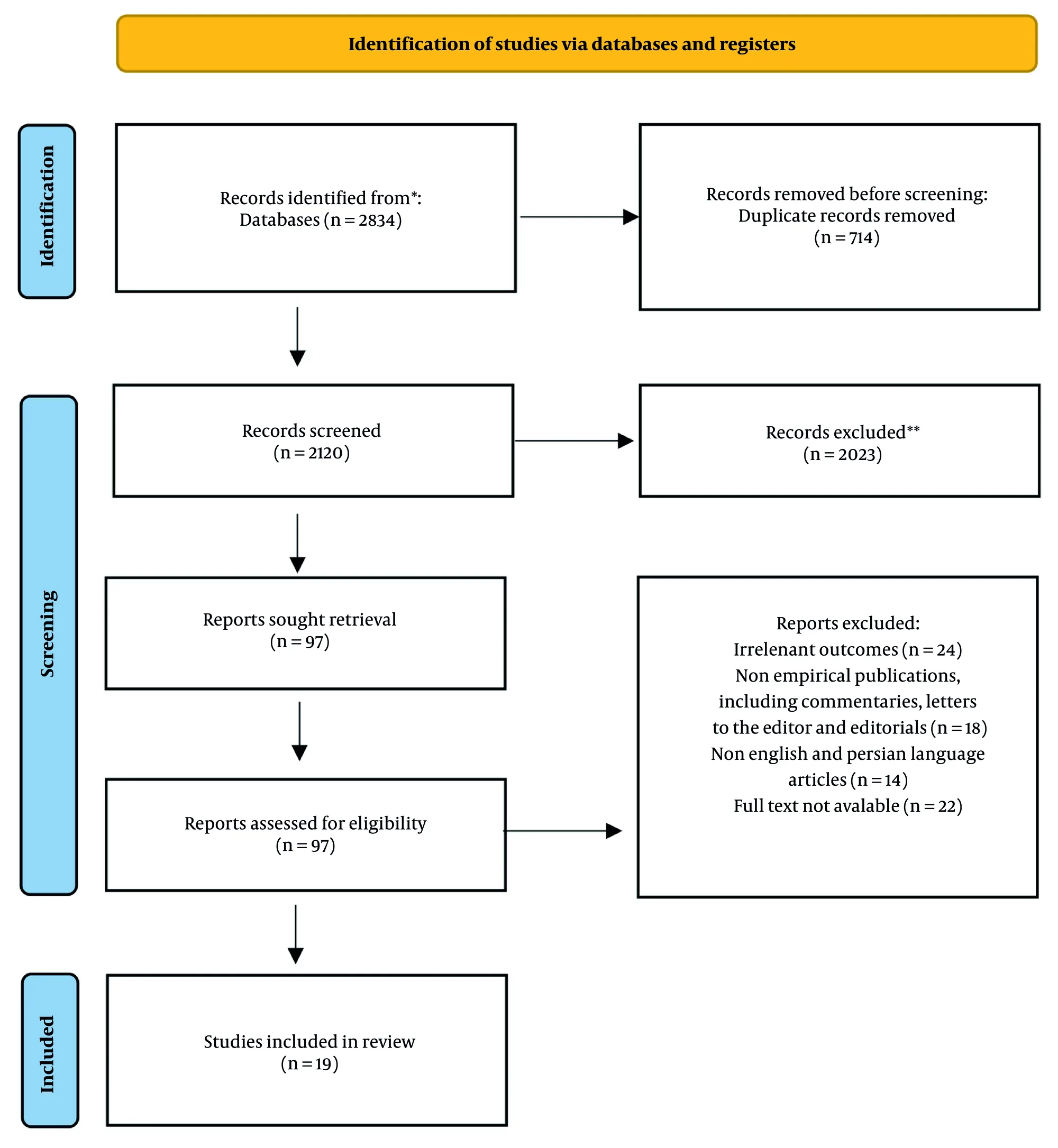
PRISMA flow diagram

This scoping review comprehensively identified and critically evaluated AI-driven digital tools designed to support diabetes self-management in children. The review followed the Preferred Reporting Items for Systematic Reviews and Meta-Analyses (PRISMA) guidelines (Figure 1) (10), ensuring a structured, transparent, and reproducible process encompassing literature searching, study selection, and data synthesis.

A comprehensive search was conducted in PubMed, Scopus, Web of Science, CINAHL, PsycINFO, and Embase to identify studies on AI-driven digital tools for pediatric diabetes self-management. The final search was completed on March 1, 2025. Search terms included Medical Subject Headings (MeSH) and relevant free-text keywords, and studies published from January 2010 through March 1, 2025, were considered. Only English-language studies were eligible (Table 1). All retrieved studies were independently screened by two reviewers to enhance accuracy and reduce selection bias. Relevant data were systematically extracted and summarized according to predefined criteria (Table 2).

**Table 1.** Databases and Search Strategies

Databases	Search Strategy
**Scopus, CINAHL, PsycINFO**	(Artificial Intelligence, Machine Learning, Deep Learning*), (Mobile Applications, Telemedicine, Remote Consultation, Wearable Electronic Devices*), (Diabetes Mellitus, Diabetes Complications*), (Child, Adolescent, Pediatrics*) (combined using AND and OR operators)
**PubMed and Web of Science**	("Artificial Intelligence"[Mesh] OR "Machine Learning"[Mesh] OR "Deep Learning"[Mesh]) AND ("Mobile Applications"[Mesh] OR "Telemedicine"[Mesh] OR "Remote Consultation"[Mesh] OR "Wearable Electronic Devices"[Mesh]) AND ("Diabetes Mellitus"[Mesh] OR "Diabetes Complications"[Mesh]) AND ("Child"[Mesh] OR "Adolescent"[Mesh] OR "Pediatrics"[Mesh])
**Embase**	"Artificial Intelligence", "Machine Learning", "Deep Learning", "Mobile Applications", "Telemedicine", "Remote Consultation", "Wearable Electronic Devices", "Diabetes Mellitus", "Diabetes Complications", "Child", "Adolescent", "Pediatrics"

**Table 2.** Characteristics and Thematic Classification of Included Studies

Year	First Author	Study Type	Sampling Method	Target population	Population Type	Participants	Study Goal	Method Used	Risk of Bias Tool	Bias Level	Key Findings
2020	Andersen (12)	Qualitative	Purposive	Children with type 1 diabetes and parents	Pediatric-only	≈ 15 - 20	To identify the needs of children with diabetes for self-management and design a digital tool concept	Conceptual design and user needs analysis	CASP	Moderate–High	Confirmed the need for personalized, interactive self-management tools
2018	Contreras (13)	Systematic Review	Systematic database search	Studies on type 1 and type 2 diabetes	Mixed-age/general diabetes population	141	To review AI applications in diabetes management and decision support	Machine learning, predictive algorithms	AMSTAR 2	Low	AI shows strong potential for glucose prediction and clinical decision support
2021	Persson (14)	Integrative Literature Review	Targeted selection of relevant studies	Type 1 and type 2 diabetes patients	Adult and mixed-age diabetes populations	38	To integrate evidence on AI use for self-care in Type 1 and Type 2 diabetes	Decision-support systems, mobile apps, deep learning	JBI Checklist	Moderate	AI improves self-care but requires more clinical validation
2022	Alaslawi (15)	Systematic Review	Systematic database search	Diabetes app users	Mixed-age diabetes app users	30	To identify factors influencing adoption of diabetes self-management apps	Mobile health (mHealth) apps	AMSTAR 2	Moderate	Ease of use and trust are key determinants of app adoption
2024	Kerth (16)	Systematic Review	Systematic search	Children/adolescents with chronic conditions	Broader pediatric chronic disease population	25	To review AI use in care of children/adolescents with chronic diseases	AI in diagnosis and self-management	ROBIS	Low	AI shows high potential for improving care and reducing caregiver burden
2023	Ansari (17)	Cross-sectional	Simple random	type 2 diabetes patients	Adult Type 2 diabetes population	120	To apply AI in assessing self-management behaviors in type 2 diabetes	Behavioral classification algorithms	ROBINS-I	Moderate	AI successfully identified weak self-management behavior patterns
2023	Neamtu (18)	Observational	Convenience	Children with type 1 diabetes	Pediatric-only	50	To predict glycemic control in children with type 1 diabetes using ML	Random Forest, SVM algorithms	ROBINS-I	Low-moderate	ML models accurately predicted HbA1c levels
2022	Zhang (19)	Observational	Voluntary sampling via app users	type 1 diabetes patients	Mixed-age Type 1 diabetes population	80	To identify self-management barriers via momentary assessment & ML	Ecological Momentary Assessment + ML	ROBINS-I	Moderate	ML identified behavioral factors affecting glucose control
2022	Campanella (20)	Experimental/analytical	Voluntary sampling	Children and adolescents with type 1 diabetes	Pediatric-only	40	To use ML to improve care in children using hybrid closed-loop systems	ML analysis of insulin pump data	RoB 2	Low	Improved glucose control and reduced hypoglycemia reported
2022	Colmegna (21)	Pilot Study	Voluntary sampling	Adults with type 1 diabetes	Mixed-age Type 1 diabetes population	25	To evaluate a web-based simulation tool for self-management in type 1 diabetes	Interactive web-based simulation tool	RoB 2	Moderate	Tool increased patients’ awareness of glucose control
2024	Colmegna (22)	Pilot Study	Purposive	type 1 diabetes patients	Mixed-age Type 1 diabetes population	12	To analyze human-machine co-adaptation in behavioral control of type 1 diabetes	Adaptive behavioral control + ML	RoB 2	Moderate	Human–machine interaction improved self-regulation and glucose control
2023	Chan (23)	Development & Validation	Dataset sampling	Patients with type 1 diabetes	Mixed-age Type 1 diabetes population	351	A machine learning framework identifies blood glucose patterns for clearer diabetes management.	CGM data + ML (dynamic time warping + clustering)	ROBINS-I	Moderate	ML framework identified 6 glucose fluctuation patterns & 4 patient clusters
2020	Nimri (24)	Randomized Controlled Trial	Random	Youths aged 10 - 21 with type 1 diabetes using pump therapy	Pediatric-only	108	AI system optimizes insulin dosing in youths with type 1 diabetes.	AI-Decision Support System for insulin adjustment	RoB 2	Low	AI-DSS non-inferior to physician-guided insulin adjustments; safe
2024	Zhang (25)	Systematic Review & Meta-Analysis	Aggregated RCTs	Children and adolescents with type 1 diabetes	Pediatric-only	1704	To evaluate telemedicine in improving glycemic control among children/adolescents with type 1 diabetes	Telemedicine interventions	AMSTAR 2	Low-moderate	Telemedicine reduced HbA1c by ~0.22%; improved quality of life modestly
2025	Elmotia (26)	Systematic Review & Meta-Analysis	Aggregated RCTs	Patients with type 1 or type 2 diabetes	Mixed-age/general diabetes population	1242	To assess effectiveness of AI-driven interventions in glycemic control	AI-driven interventions	AMSTAR 2	Low-moderate	AI interventions improve glycemic outcomes; heterogeneity high
2024	Sherazi (27)	Cross-Sectional	Usability sampling	Children aged ≤ 12 years with type 1 diabetes	Pediatric-focused/mixed adolescent population	Not applicable	To evaluate usability and satisfaction with mHealth app in rural and remote areas	mHealth app for type 1 diabetes care	ROBINS-I	Moderate	mHealth can enhance access & satisfaction in remote areas
2025	Aguayo (28)	Randomized Controlled Trial	Random	Children and adolescents with type 1 diabetes	Pediatric-only	27	Evaluate Diactive-1 usability in youth with type 1 diabetes	Diactive-1 mHealth intervention	RoB 2	Moderate	Personalized mHealth for exercise + education feasible in youth
2022	Schmidt (29)	Design Study / Intervention Development	Design sampling	Adolescents with type 1 diabetes	Pediatric-only	Not applicable	Design an mHealth self-management learning experience for adolescents with type 1 diabetes	mHealth self-management intervention	ROBINS-I	Moderate	Design emphasizes engagement and educational tailoring for adolescents
2025	Yousif (30)	Systematic Review	Literature search	Pediatric diabetes patients ( < 18)	Pediatric-only	10	Assess transparency and validity of AI applications in pediatric diabetes	AI applications in pediatric diabetes	AMSTAR 2	Moderate	Only 60% disclosed algorithm details; transparency & validity issues remain

### 2.2. Eligibility Framework

Population (P): Children and adolescents aged 0 - 18 years with type 1 or type 2 diabetes mellitus.

Intervention (I): AI-driven digital tools supporting diabetes self-management, including mobile health applications, decision-support systems, telemedicine platforms, wearable technologies, and machine learning-based interventions.

Comparator (C): Standard care, non-AI interventions, physician-led management, or baseline conditions, when applicable. A comparator was not required for inclusion.

Outcomes (O): Glycemic outcomes, self-management behaviors, usability, engagement, quality of life, accessibility, and technological performance.

### 2.3. Search Strategy

A comprehensive literature search was conducted across six major electronic databases: PubMed, Scopus, Web of Science, CINAHL, PsycINFO, and Embase. These databases were selected for their broad coverage of health, medical, and social science research. Equivalent search concepts and database-specific indexing terms were adapted for Scopus, Web of Science, Embase, CINAHL, and PsycINFO. Searches were limited to studies published from January 2010 through March 1, 2025. The reference lists of included studies and relevant reviews were also manually screened to identify additional eligible publications.

PubMed search strategy:

(("Artificial Intelligence"[Mesh] OR "Machine Learning"[Mesh] OR "Deep Learning"[Mesh] OR artificial intelligence[tiab] OR machine learning[tiab] OR deep learning[tiab]) AND ("Diabetes Mellitus"[Mesh] OR diabetes mellitus[tiab] OR type 1 diabetes[tiab] OR type 2 diabetes[tiab]) AND ("Child"[Mesh] OR "Adolescent"[Mesh] OR child*[tiab] OR pediatric*[tiab] OR adolescen*[tiab]) AND ("Self-Management"[Mesh] OR self-management[tiab] OR self care[tiab]) AND ("Mobile Applications"[Mesh] OR telemedicine[tiab] OR digital health[tiab] OR mHealth[tiab] OR wearable devices[tiab]))

### 2.4. Inclusion and Exclusion Criteria

Studies were included if they met the following criteria:

Focus: Children and adolescents aged 0 - 18 years with type 1 or type 2 diabetes mellitus using AI-driven digital tools for self-management.

Study Design: Eligible studies evaluated AI-driven digital tools designed to support diabetes self-management in children and adolescents aged 0 - 18 years with type 1 or type 2 diabetes mellitus. Eligible designs included randomized controlled trials, quasi-experimental studies, observational studies, qualitative studies, pilot studies, systematic reviews, and meta-analyses relevant to pediatric diabetes self-management technologies.

Outcomes: Studies reported clinical outcomes; self-management behaviors, including adherence to therapy, diet, and physical activity; usability and engagement, including acceptability and satisfaction; quality of life; or technological features of AI tools, including predictive analytics, personalization, and feedback.

Timeframe: Studies published between 2010 and March 1, 2025.

Language: English-language publications only.

Studies were excluded if they:

1) Focused exclusively on adults or mixed populations without separate pediatric data.

2) Investigated digital tools not powered by AI or general educational interventions without AI components.

3) Were narrative reviews, editorials, commentaries, conference abstracts, protocols, or non-empirical conceptual papers. To minimize duplication of evidence, primary empirical studies and secondary evidence sources were synthesized separately during data analysis.

4) Were published in languages other than English.

5) Lacked sufficient information on intervention features or outcomes related to diabetes self-management.

These criteria ensured that only studies directly relevant to AI-driven digital tools for pediatric diabetes self-management were included, providing a focused and comprehensive synthesis of the available evidence.

### 2.5. Data Extraction and Management

Data extraction was performed independently by two reviewers using a standardized form. Extracted information included:

Study Characteristics: Author(s), year, study design, sample size, and setting.

Intervention Characteristics: Type and format of AI-driven digital tools (e.g., mobile apps, wearable devices, telemedicine), duration, frequency, and targeted diabetes self-management tasks.

Outcomes: Clinical outcomes (HbA1c, glucose monitoring), self-management behaviors, usability and engagement, quality of life, and technological features of AI tools.

Methodological Details: Assessment tools (surveys, interviews, knowledge tests) and analytical approaches.

Initial reference records were organized in Google Sheets and subsequently managed in EndNote to prevent duplication and maintain accurate citation tracking. The search strategy was developed by the lead author and refined in collaboration with two senior team members with expertise in systematic text synthesis. Although a formal librarian was not involved, the final search strategy underwent internal review to ensure comprehensiveness. Disagreements regarding study selection or data extraction were resolved through discussion or, when necessary, consultation with a third-party adjudicator. These procedures were implemented to maintain consistency, transparency, and methodological rigor throughout the review process.

### 2.6. Quality Assessment

Given the methodological diversity of the included studies, multiple validated appraisal tools were applied according to study design. Randomized controlled trials were assessed using the Cochrane Risk of Bias 2 (RoB 2) tool. Non-randomized interventional and observational studies were evaluated using ROBINS-I or the Newcastle-Ottawa Scale (NOS), depending on study structure and outcome reporting. Qualitative studies were appraised using the CASP checklist. Systematic reviews and meta-analyses were assessed using AMSTAR 2 and ROBIS to evaluate methodological rigor and review-level bias. Integrative and mixed-methods reviews were appraised using Joanna Briggs Institute (JBI) critical appraisal tools, where applicable. QUADAS-2 was applied only to studies involving diagnostic or predictive modeling performance assessment. Narrative reviews, commentaries, editorials, and conceptual non-empirical papers were not subjected to formal risk-of-bias assessment because validated appraisal frameworks for these publication types are limited. Following methodological reassessment, these publication types were excluded from the final synthesis. Quality assessment was performed independently by two reviewers, with disagreements resolved through discussion. No studies were excluded solely on the basis of quality ratings; however, studies judged to have a moderate or high risk of bias were interpreted cautiously during the narrative synthesis and discussion of findings.

### 2.7. Data Synthesis

Because of heterogeneity in study designs and outcome measures, a narrative thematic synthesis was conducted according to the six-step framework of Braun and Clarke (11), including data familiarization, coding, theme development, theme review, theme definition, and reporting. Two reviewers independently performed inductive coding of all included studies, with disagreements resolved through discussion or consultation with a third reviewer. Recurring patterns were organized into thematic categories addressing accessibility, flexibility, knowledge retention, patient engagement, and the complementary role of traditional teaching methods, alongside challenges such as inconsistent content quality, limited technological access, and demographic disparities. Findings were synthesized narratively to highlight the benefits and limitations of AI-based digital tools for pediatric diabetes self-management. To minimize duplication and overrepresentation, primary studies and secondary evidence sources were analyzed separately, whereas narrative reviews, commentaries, and conceptual non-empirical papers were excluded following methodological reassessment.

## 3. Results

### 3.1. Study Selection and Included Evidence

Following a rigorous screening process, 19 articles were eligible for inclusion. The initial search of six databases identified studies investigating AI-based digital tools for diabetes self-management in children. The study-selection process is presented in a PRISMA-compliant flow diagram (Figure 1), which shows the numbers of records identified, screened, assessed for eligibility, and included, along with reasons for exclusion. The characteristics of the included studies are presented in Table 2. Of the 19 included studies, 11 were primary empirical investigations, including randomized controlled trials, observational studies, qualitative studies, and pilot studies, whereas 8 were secondary evidence sources, including systematic reviews, meta-analyses, and integrative reviews. To minimize duplication of evidence, primary and secondary studies were synthesized separately. Primary studies informed conclusions regarding clinical effectiveness and usability, whereas review-based studies were used primarily to contextualize broader trends, implementation challenges, and future research priorities.

### 3.2. Application Domains

The 19 included studies addressed several overlapping application domains of AI-driven digital tools for pediatric diabetes self-management. Overall, 8 studies focused primarily on glucose prediction, glycemic monitoring, or pattern recognition; 4 studies evaluated insulin-dosing optimization and clinical decision-support systems; 6 studies investigated personalized education, behavioral support, user engagement, or self-management learning interventions; 5 studies examined telemedicine, remote monitoring, or healthcare accessibility; and 5 studies focused primarily on ethical, methodological, transparency, or implementation challenges.

### 3.3. Population Characteristics of Included Studies

Most included studies focused primarily on children and adolescents with type 1 diabetes mellitus. However, several studies involved mixed-age populations, adult participants, or broader chronic-disease and digital-health contexts. To improve interpretability and applicability, findings were synthesized according to four population categories: 1) pediatric-only diabetes studies; 2) mixed-age diabetes studies; 3) adult-focused diabetes studies; and 4) broader contextual AI-healthcare literature. The primary interpretation of clinical effectiveness, usability, and self-management outcomes was based predominantly on pediatric empirical studies. Broader adult and conceptual literature was used mainly to contextualize implementation challenges, ethical considerations, and future research directions.

Several included studies reported measurable improvements in diabetes-management outcomes. Machine learning-based models demonstrated good performance in predicting glycemic control and identifying glucose-fluctuation patterns. AI-supported decision systems were reported to be safe and comparable to physician-guided insulin adjustment in youth with type 1 diabetes. Telemedicine interventions showed modest reductions in HbA1c and improvements in quality of life. In addition, mHealth-based interventions generally reported high levels of usability, satisfaction, and user engagement. However, the magnitude of clinical benefit varied across studies, and many investigations focused primarily on feasibility, usability, or system development rather than long-term clinical outcomes.

### 3.4. Thematic Synthesis of Findings

Thematic analysis identified four major themes and associated subthemes related to AI-driven digital tools for pediatric diabetes self-management.

#### 3.4.1. Theme 1: Clinical Effectiveness and Glycemic Outcomes

Subthemes included HbA1c improvement, reduction in hypoglycemia episodes, enhanced glucose prediction and monitoring, and optimization of insulin dosing. This theme was primarily supported by randomized controlled trials, observational studies, and machine learning-based intervention studies involving pediatric type 1 diabetes populations. Key contributing studies included Chan et al., Bratke et al., Schoelwer et al., Fleming et al., Shan et al., Heintzman, and Garabedian et al. (23, 31-36).

#### 3.4.2. Theme 2: Personalization, Engagement, and Behavioral Support

Subthemes included personalized feedback systems, gamification and motivation, user-centered educational support, and parent-child interaction and caregiver involvement. Evidence for this theme emerged mainly from qualitative studies, usability studies, and pediatric mHealth interventions. Nimri et al. and Zhang et al. contributed substantially to this category (24, 25).

#### 3.4.3. Theme 3: Usability, Accessibility, and Technology Integration

Subthemes included ease of use, integration with clinical workflows, remote monitoring capabilities, and accessibility in rural and underserved settings. Findings were supported primarily by observational studies, implementation studies, and telemedicine-focused reviews (26).

#### 3.4.4. Theme 4: Ethical, Privacy, and Methodological Challenges

Subthemes included data privacy concerns, algorithm transparency, clinical accountability, limited long-term validation, and methodological heterogeneity. This theme was informed mainly by systematic reviews, methodological analyses, and ethical discussions, including studies by Sherazi et al., Aguayo et al., Schmidt et al., Yousif et al., and Chng et al. (27-30, 37).

Across themes, the quality of evidence varied substantially. Most pediatric primary studies involved relatively small sample sizes, short follow-up periods, and heterogeneous intervention designs. Therefore, findings related to long-term effectiveness, scalability, and clinical implementation should be interpreted cautiously.

## 4. Discussion

This scoping review synthesized current evidence regarding artificial intelligence (AI)-driven digital tools designed to support diabetes self-management in children and adolescents. The findings indicate that AI technologies are increasingly being integrated into pediatric diabetes care through applications such as glucose-prediction systems, insulin-dose decision-support platforms, telemedicine services, adaptive learning environments, and personalized self-management interventions. Collectively, these technologies have the potential to enhance disease monitoring, facilitate individualized care, and strengthen patient engagement. However, the overall evidence base remains heterogeneous and should be interpreted with caution. One of the most consistent observations across the included studies was the ability of AI-enabled systems to manage large and complex streams of diabetes-related data. Machine learning algorithms showed particular promise in identifying glycemic patterns, predicting glucose fluctuations, and supporting insulin-adjustment decisions. These capabilities are especially relevant in pediatric diabetes, where daily management requires continuous interpretation of dynamic physiological and behavioral information. By transforming large volumes of patient-generated data into actionable recommendations, AI-based systems may reduce the cognitive burden for both children and caregivers while supporting more timely and individualized decision-making (21, 23, 24, 26, 38).

Beyond glycemic management, the reviewed literature highlighted the growing role of AI in promoting behavioral engagement and self-management support. Several interventions incorporated personalized feedback mechanisms, adaptive educational content, remote communication features, and user-centered design principles. Such approaches may be particularly valuable during childhood and adolescence, developmental periods characterized by evolving autonomy, fluctuating motivation, and increasing responsibility for disease management. The findings suggest that successful interventions extend beyond technological functionality and often depend on alignment with developmental needs, family involvement, and everyday clinical workflows (12, 15, 27, 29). Despite these encouraging findings, the evidence was not uniformly positive. A substantial proportion of included studies did not directly evaluate clinical effectiveness and instead focused on usability, feasibility, acceptability, system design, or implementation processes. Although these studies generally reported favorable user experiences and high satisfaction, they provided limited evidence of meaningful improvements in glycemic outcomes or long-term diabetes control. Consequently, positive perceptions of AI-based tools should not automatically be interpreted as evidence of clinical benefit (28, 29, 32). Furthermore, several studies reported modest rather than substantial improvements in diabetes outcomes. Although reductions in HbA1c and improvements in self-management behaviors were observed in some investigations, the magnitude of these effects varied considerably across studies. Differences in intervention design, follow-up duration, target populations, outcome measures, and healthcare settings likely contributed to this variability. Therefore, direct comparisons between studies remain difficult, and current evidence does not support definitive conclusions regarding the superiority of any specific AI approach (22).

Compared with previous literature, this review extends current understanding in several ways. Earlier studies largely focused on physiological monitoring and predictive modeling (16, 39). An important finding of this review is the distinction between algorithmic performance and real-world effectiveness. Many studies demonstrated promising predictive accuracy or technical performance under controlled research conditions. However, relatively few investigations assessed whether these technological advances translated into sustained improvements in everyday self-management, treatment adherence, quality of life, or long-term metabolic outcomes. High predictive accuracy alone does not necessarily guarantee clinical utility. Future research should therefore prioritize patient-centered outcomes alongside technical performance metrics to determine whether algorithmic innovations produce meaningful benefits in routine clinical practice (17-20).

Another noteworthy observation is the apparent scarcity of explicitly negative findings in the literature. None of the included studies reported clear evidence that AI interventions worsened clinical outcomes; however, the absence of negative findings should not be interpreted as confirmation of effectiveness. Publication bias may contribute to the predominance of favorable reports, particularly in rapidly evolving technological fields in which innovative interventions are more likely to be published when positive outcomes are observed. Moreover, studies with insufficient statistical power may fail to detect adverse or null effects. Therefore, the current literature may overestimate the benefits of AI-based approaches, underscoring the importance of independent validation and rigorous long-term evaluation (25, 30, 37). In addition, many interventions have been trialed in high-resource settings, highlighting the need for culturally and socioeconomically adaptable solutions to ensure equitable access (40).

Taken together, these findings suggest that AI-driven digital tools represent a promising but still developing component of pediatric diabetes self-management. Current evidence supports their potential to enhance monitoring, decision support, patient engagement, and personalized care. Nevertheless, important uncertainties remain regarding long-term effectiveness, clinical impact, scalability, equity, and implementation. Future research should prioritize adequately powered randomized controlled trials, standardized outcome reporting, transparent algorithm-validation procedures, and long-term assessments conducted in diverse pediatric populations. Addressing these priorities will be essential to determine whether the promise of AI can be translated into sustainable improvements in diabetes outcomes for children and adolescents.

### 4.1. Implications for Practice and Future Research

AI-powered digital solutions may improve daily diabetes care for children by supporting parents and caregivers in making well-informed decisions. These technologies can enable continuous glucose tracking, provide medication reminders, and offer personalized educational content, thereby reducing caregiver burden. Effective use should account for the child’s developmental stage, accessibility, and family acceptance. Future research should examine the long-term effects of these AI tools across diverse populations to improve generalizability. Additional studies are needed to address data accuracy and privacy concerns and to refine AI algorithms. Randomized controlled trials and analyses of caregiver-system interactions may provide stronger evidence of effectiveness.

### 4.2. Conclusions

This scoping review identified a growing body of evidence supporting the use of AI-driven digital tools in pediatric diabetes self-management. Across the included studies, AI applications showed potential to improve glucose monitoring, glycemic prediction, insulin-dose optimization, remote-care delivery, and user engagement. However, the available evidence remains limited by methodological heterogeneity, small sample sizes, and short follow-up periods. Although the findings suggest that AI-based interventions may enhance self-management and support clinical decision-making in children and adolescents with diabetes, stronger pediatric-focused studies are needed to establish their long-term effectiveness, safety, transparency, and real-world applicability.

### 4.3. Study Limitations

This scoping review has several limitations. A formal search of grey-literature sources was not conducted as part of the original review protocol. The included studies often focused on specific age groups or populations, limiting the generalizability of findings to all children with diabetes. Most studies were short term and varied in design and quality, making direct comparisons difficult. Only English-language publications were considered, potentially excluding relevant research in other languages. Finally, many interventions relied on self-reported data from children or caregivers, which may introduce reporting bias, and the long-term effectiveness of AI-driven digital tools remains unclear.

## Data Availability

The dataset presented in the study is available on request from the corresponding author during submission or after publication.
